# Finishing stationary cycling too early after anterior cruciate ligament reconstruction is likely to lead to higher failure

**DOI:** 10.1186/s13102-021-00377-y

**Published:** 2021-11-25

**Authors:** Balázs Sonkodi, Endre Varga, László Hangody, Gyula Poór, István Berkes

**Affiliations:** 1 Department of Health Sciences and Sport Medicine, University of Physical Education, Budapest, Hungary; 2 Department of Traumatology, University of Szeged, Szeged, Hungary; 3 Department of Traumatology, Semmelweis University, Budapest, Hungary; 4 National Institute of Musculoskeletal Diseases, Budapest, Hungary; 5 Semmelweis University Medical School, Budapest, Hungary

**Keywords:** Anterior cruciate ligament reconstruction, Rehabilitation, Anterior cruciate ligament re-injury, Early osteoarthritis, Stationary cycling

## Abstract

**Background:**

Anterior cruciate ligament injury arises when the knee anterior ligament fibers are stretched, partially torn, or completely torn. Operated patients either end up re-injuring their reconstructed anterior cruciate ligament or majority develop early osteoarthritis regardless of the remarkable improvements of surgical techniques and the widely available rehabilitation best practices. New mechanism theories of non-contact anterior cruciate ligament injury and delayed onset muscle soreness could provide a novel perspective how to respond to this clinical challenge.

**Main body:**

A tri-phasic injury model is proposed for these non-contact injuries. Mechano-energetic microdamage of the proprioceptive sensory nerve terminals is suggested to be the first-phase injury that is followed by a harsher tissue damage in the second phase. The longitudinal dimension is the third phase and that is the equivalent of the repeated bout effect of delayed onset muscle soreness. Current paper puts this longitudinal injury phase into perspective as the phase when the long-term memory consolidation and reconsolidation of this learning related neuronal injury evolves and the phase when the extent of the neuronal regeneration is determined. Reinstating the mitochondrial energy supply and ‘breathing capacity’ of the injured proprioceptive sensory neurons during this period is emphasized, as avoiding fatigue, overuse, overload and re-injury.

**Conclusions:**

Extended use, minimum up to a year or even longer, of a current rehabilitation technique, namely moderate intensity low resistance stationary cycling, is recommended preferably at the end of the day. This exercise therapeutic strategy should be a supplementation to the currently used rehabilitation best practices as a knee anti-aging maintenance effort.

## Background

Anterior cruciate ligament (ACL) injury arises when the knee anterior ligament fibers are stretched, partially torn, or completely torn. The average annual increase of ACL injuries has shown a gradual increase in the past two decades among collegiate athletes [1], but similar increasing trend could be the case in the non-athlete population as well. As a result, the number of anterior cruciate ligament reconstruction (ACLR) surgeries has been on the rise in the past decades. The surgical techniques of ACLR have gone through remarkable improvements during this period of time, not to mention the widely available effective rehabilitation best practices. Nevertheless, something is missing from current rehabilitation practices, because emerging evidence supports that majority of operated patients either end up re-injuring their reconstructed ACL, or they develop early aging in the form of early osteoarthritis (OA) [2]. Noteworthy, there has been even an estimate that an ACL rupture is accelerating the aging of the knee by 30 years [3]. New theories [4, 5] could provide novel perspective how to fill in a supplemental puzzle piece in rehabilitation practices after ACLR.

## Main text

### Injury mechanism and the critical role of proprioceptive sensory neurons

Non-contact ACL injury, involving around 80% of ACL injuries [1, 6–11], is theorized to be a result of an acute proprioceptive axon terminal mechano-energetic microdamage in the proximal tibia in unaccustomed or strenuous eccentric exercise moments [5]. This peripheral proprioceptive neuronal microlesion could evolve into a bi-phasic injury mechanism, where the primary proprioceptive impairment could result in a secondary harsher tissue injury and that is when the ACL injury could also prevail [5]. The primary injury is theorized to be caused by the acute compression microlesion of the proprioceptive sensory terminals at the proximal tibia [5]. Part of the hypothesis that these sensory nerves contribute to the stretch reflex at the spinal dorsal horn [5].

Accordingly, this proprioceptive neuronal impairment is suggested to alter the static encoding of the affected stretch reflex in order to enhance postural control, enhance anti-gravitational capacity and to enhance shock attenuation [5, 12]. Spinal and supraspinal changes in the nervous system due to ACL injury is either established or suspected [2, 12]. Noteworthy, that the same non-contact bi-phasic injury and compensatory mechanism is hypothesized in delayed onset muscle soreness (DOMS) as well [4]. Moreover, there is evidence that DOMS not only affects agonist muscles, but ipsilateral antagonist as well, not to mention the contralateral effect [13–15]. These findings not only imply the involvement of the stretch reflex, but a preprogrammed and orchestrated secondary spinal compensatory mechanism [5, 12, 16] that certainly alters supraspinal mechanism as well.

It is notable that very small area, approximately 1%, of ACL consists of proprioceptors and some of them contribute to the position sense of the knee joint [17–19]. Accordingly, the clinical utility of the injured ACL in ACLR has shown better results [20–22]. More importantly, the findings that a more extensive bone bruise prevails as a secondary damage [23], due to the hypothesized primary injury [5], also could imply further compression or “double crush” proprioceptive neuronal damage [5]. Finally, it’s worth to mention that there is a potential risk of even further neuronal injury of the infrapatellar branch of the saphenous nerve during ACLR, when harvesting of autologous tendons from the gracilis and semitendinosus muscles is executed [24–28]. The infrapatellar branch of the saphenous nerve is implicated even in the primary injury mechanism of non-contact ACL injury as a nerve that could innervate the periosteum of the proximal tibia with proprioceptive contribution to the stretch reflex [5].

### Repeated bout effect and memory consolidation

We could possibly learn from the longitudinal dimension of DOMS and that is the repeated bout effect (RBE). Initial bout of severe DOMS-inducing unaccustomed exercise entailing eccentric contractions could be evoked for at least 6 months, but is lost between 9 and 12 months [12, 29, 30]. Moreover, the repeated bout effect could be induced on the contralateral side as well [31–33]. We could translate these findings as, if the preprogrammed postural control encoding is impaired by this learning related proprioceptive terminal microdamage or axonal injury, then a protective, but less efficient and energy consuming preprogrammed secondary compensatory messenger pathway is activated on the spinal dorsal horn with a long-term memory consolidation process [12, 16]. Noteworthy, that these type of impairment is suggested to be a terminal arbor degeneration (TAD) like lesion at the proprioceptive sensory terminal and does not come with a classical Wallerian type neuronal degeneration process [4]. Furthermore, once full functional regeneration of this TAD like lesions occur then the long-term memory will be extinct between 9 and 12 months. Important to note, that RBE is considered to be a protective mechanism [34].

Memory consolidation is a lengthy, time-dependent process leading to altered and strengthened synaptic connections between neurons. The memory dimensions of these proprioceptive TAD like lesions entail several memory pathways, like short-term working memory, long-term episodic memory, inflammation and pain memory [12]. Noteworthy, that acute compression proprioceptive sensory axonopathy induced activated N-methyl-D-aspartate (NMDA) receptors are suspected as the primary gate controllers of these memory acquisition processes [5, 12].

Overall, we have every ground to suspect that ACL injuries have their own RBE that involves for example the initiation of fear memory consolidation. The lack of extinction of this RBE protective mechanism without the full functional regeneration of these proprioceptive neurons could elongate this ‘third injury phase’ with facilitated long-term memory reconsolidation and accelerated aging. Strategic focus on the maximization of the functional regeneration of these injured peripheral proprioceptive sensory neurons and the minimization of their long-term memory consolidation processes, including fear memory, is what seems to be the missing link.

### Cycling and neuronal regeneration

An earlier finding is that long-term or extended, but light to moderate concentric exercise could alleviate the symptoms of the chronic dimension of this type of proprioceptive terminal microdamage or axonal injury [4, 35]. These mechano-energetic TAD like lesions seem to be the consequence of an acute stress reaction (ASR) induced mitochondrial energy depletion and mechanical Piezo2 ion channel microdamage, happening in unaccustomed or strenuous eccentric, learning related, exercise moments [4, 12, 36]. Therefore, the proposed strategy is to keep the mitochondrial ‘breathing capacity’ of these impaired proprioceptive axon terminals and axons in good shape or even enhance it with concentric training for at least a year or even longer, otherwise their functional regeneration could be compromised and the longitudinal memory consolidation will be facilitated. By doing so, we could promote a more optimal rehabilitation environment for these impaired proprioceptive terminals/axons and eventually we could prevent reconsolidation or even promote extinction of injury related memory pathways.

Important to emphasize the significance of these proprioceptive sensory neurons, because they are suggested to be guiding growth, regeneration and remodelling [4]. Current authors propose that the loss of proprioceptive regeneration and remodelling capacity could lead to the ‘third injury phase’ or to earlier aging in the form of OA, due to impaired proprioceptive afferent signalling to the central nervous system. Therefore, the extent of lost functional proprioceptive sensory capacity could matter in regards to long-term outcome.

Moderate evidence is highlighting that the quadriceps torque variability increases over time after ACLR [37, 38]. Moreover, increased torque variability could be also observed in osteoarthritic patients [39]. Tayfur et al. [38] interpreted these findings as the long-term neuromuscular alteration of the quadriceps motor control could be one factor that could potentially contribute to the onset of knee OA. Current authors are even suggesting that the impaired proprioceptive sensory feedback could lead to the impaired muscle control proposed by Tayfur et al. [38] Noteworthy, that the consequence of TAD like lesions of proprioceptive termnals is theorized to be inward currents induced exaggerated contractions and that could eventually contribute to the non-contact injury of the ACL [5]. Furthermore, arthrogenic muscle inhibition evolves as a consequence of ACL injury which is theorized to be part of a preprogrammed protective secondary compensatory microcircuit [5]. Moderate evidence is showing there is no change in cortical excitability or in spinal-reflex excitability in the short-term after ACL injury [38]. However, strong evidence is showing that cortical excitability is decreasing and spinal-reflex excitability is increasing in the longer term [38]. This long-term alteration of excitability is suggested by the current authors to be due to the impaired proprioceptive sensory feedback as well. It has been postulated that the precise control of movement is crucial for knee function [37, 38]. The impaired control may lead to alterations in joint loading and eventually could lead to degenerative cartilage changes [37, 38, 40]. Onate et al. [41] used the analogy for ACLR as a “torn electrical cord is appropriately put back together, but the cord does not properly conduct electricity in its previous fashion”.

Essential part of the recommended strategy is to avoid further proprioceptive sensory injury, fatigue, overuse and overloading, especially up to at least one year after ACLR, because they could facilitate the compensatory secondary microcircuits with concomitant low-grade neuroinflammation and detrimental facilitation of long-term memory reconsolidation [5]. Good news that these peripheral nerves have a great affinity for regeneration, but their ‘breathing capacity’ should be reinstated, maintained or even enhanced on an extended way by training and adaptation, because TAD like terminal lesions and non-contact injuries are fatigue related. It is possible that the extent of proprioceptive nerve injury due to ACL injury or ACLR surgery is so high that full functional regeneration is not feasible and in these cases the question is rightly addressed whether “does it ever get back to normal” [38]. However, even in these cases maintaining or enhancing the ‘breathing capacity’ of proprioceptive sensory neurons, besides strength and neuromuscular functional enhancement, could likely prevent reinjury or prolong the initiation of OA.

### Longitudinal phase of ACL injury and extended cycling

The incidence of second ACL injury rates are reported to be 23% [42], while early osteoarthrosis evolves in more than 4/5 of the cases after ACL injury [43]. The suggested non-pharmacological exercise therapy to prevent or delay the ‘third injury phase’ could be the extended, minimum up to a year, light to moderate home-based concentric exercise in the form of stationary bicycle training with low resistance [12]. The basis of this supplemental exercise strategy is threefold. First, concentric exercise enhances aerobic capacity, therefore the mitochondrial ‘breathing capacity’ of the impaired proprioceptive neurons could be maintained or even boosted. Second, exercise with the circumvention of proprioceptive loading is hypothesised to even promote proprioceptive neuronal regeneration [12]. Third, we could attenuate supraspinal loading with low resistance stationary biking [12].

Important to note, that proprioceptive loading has two dimensions after ACLR. First, the secondary spinal compensatory mechanism as a result of the neuronal microinjury and the resultant spinal loading uses more synaptic connections which means enhanced neuro-energetic usage. This secondary compensatory mechanism is theorized to be represented in the delayed latency of the medium latency response (MLR) of the stretch reflex and affects the static encoding of the stretch reflex [5, 12]. Accordingly, there is evidence of delayed latency of MLR after ACL rupture [44]. The second dimension of proprioceptive loading is the increased amplitude of MLR and even enhanced short-latency responses due to postural threat which also requires neuro-energetic mobilization in the form of increased stretch reflex dynamic sensitivity [45]. Noteworthy, that athletes with ACLR show not only arthrogenic muscle inhibition [5, 46, 47], but increased cognitive loading on neuromuscular control [47]. The elevated cognitive loading is accredited by current authors to increased postural threat that might be attributed to the greater knee joint position sense error even after ACLR and rehabilitation [47].

Indicative from animal studies, that ASIC3 ion channels, in addition to the primary Piezo2 channels, are involved in proprioceptive mechanotransduction [48, 49]. Furthermore, it is also demonstrated that the levels of ASIC3 expression in the dorsal root ganglion of proprioceptive sensory neurons innervating the knee joint in OA rats showed gradual increase as OA progressed and they play a critical role in secondary hyperalgesia [50, 51]. Noteworthy, that ASIC3 invokes sustained inward currents and these inward currents are hypothesized to play a role in the mechanism of non-contact ACL injury and in exaggerated contractions [48]. Finally, ASIC3 channels in the brain alter acid-evoked currents that leads to fear conditioning [52]. Not surprisingly, postural threat increases the dynamic sensitivity of the stretch reflex [45].

Moreover, the two dimensions of proprioceptive loading could be interrelated through GABAergic pathways. The reduction of GABAergic inhibition in the spinal cord ventral horn could contribute to the generation of persistent inward currents and exaggerated quadriceps contractions [5, 53], as the reduction of GABAergic inhibition within the motor cortex is suspected as a cause of quadriceps arthrogenic muscle inhibition [5, 54]. In addition, GABAergic transmission has a role in acquisition, consolidation, reconsolidation and extinction of fear memory [55]. It was also demonstrated that ASICs have a role on GABAergic neuronal activity in the regulation of hippocampal neuronal activity [56], where fear memory is encoded [57]. Knee injury-related fear is considered to be a serious psychological factor inhibiting an athlete’s return to sports following ACLR [58, 59]. In fact, it could evolve into conscious “overthinking” or cognitive overloading of already learnt, routine, mainly unconscious maneuvers [60]. However, current authors argue that knee injury-related fear has its neuronal roots more on the periphery, related to the original proprioceptive microdamage, but certainly the peripheral mechano-energetic trauma gradually extend its effect to the central nervous system as suggested by Kakavas et al. [61].

Both of the above proprioceptive loading dimensions are suggested to be alleviated or circumvented by low resistance moderate intensity stationary bicycle training [12]. Moreover, it is important to note that since the primary injury is proposed to be learning and memory related [5, 12], therefore using external focus motor learning techniques in ACLR rehabilitation is preferred as oppose to internal focus of attention [2], because the altered supraspinal cognitive loading factor in the long-term memory consolidation of the primer neuronal injury is suggested to be minimized [12]. Accordingly, the theoretical basis for unloading of proprioception by “closed gate” stationary biking, which is the circumvention of central sensory-loading or supraspinal loading, is that NMDA receptors of motoneurons could actively produce intrinsic rhythmic activity along with the central pattern generators of locomotion on the spinal level [12].

In addition, the symmetric loading and cyclic feature of cycling have favourable effect on postural control and gait performance [12] since alleviates the asymmetric joint loading nature of this post-injury state [2]. It is highly important to emphasize, that following this strategy should be a supplemental exercise therapy, not alternative, of the current best practice solutions in ACLR rehabilitation, including targeting the neuromuscular control system, sport specific rehabilitation and individually tailored motor-learning techniques. Not abiding to extended cycling is hypothesised to increase the likelihood of making ACL injury related spinal and supraspinal changes permanent or even worse, progressive. Couple weeks of rehabilitative cycling with positive outcome is not enough time for the proprioceptive nerves to regenerate, maintain, not to mention enhance ‘breathing capacity’. Important to note, that the above moderate intensity low resistance stationary cycling without substantial proprioceptive loading is recommended after eccentric muscle actions [62], rehabilitation sessions and at the end of the day, but not as late to interfere with sleeping, because lack of sleeping is also a very important risk factor of neuronal regeneration.

## Conclusion

After all, the take away message is not to stop cycling too early in the rehabilitation process of ACLR, but follow up on an extended way for at least a year or even longer, as a knee anti-aging maintenance effort. It needs to be emphasized that the proposed strategy in perspective cannot hurt and is currently in use in the early stage of ACLR rehabilitation as a best practice solution, but should be prescribed in an extended way!

## Data Availability

Not applicable.

## Abbreviations

ACL: Anterior cruciate ligament
ACLR: Anterior cruciate ligament reconstruction
ASR: Acute stress reaction
DOMS: Delayed onset muscle soreness
MLR: Medium latency response
NMDA: N-methyl-D-aspartate
OA: Osteoarthritis
RBE: Repeated bout effect
TAD: Terminal arbor degeneration

## References

[1] Hootman JM, Dick R, Agel J (2007). Epidemiology of collegiate injuries for 15 sports: summary and recommendations for injury prevention initiatives. J Athl Train.

[2] Gokeler A, Neuhaus D, Benjaminse A, Grooms DR, Baumeister J (2019). Principles of motor learning to support neuroplasticity after ACL injury: implications for optimizing performance and reducing risk of second ACL injury. Sports Med.

[3] Lohmander LS, Ostenberg A, Englund M, Roos H (2004). High prevalence of knee osteoarthritis, pain, and functional limitations in female soccer players twelve years after anterior cruciate ligament injury. Arthritis Rheum.

[4] Sonkodi B, Berkes I, Koltai E (2020). Have we looked in the wrong direction for more than 100 years? Delayed onset muscle soreness is, in fact, neural microdamage rather than muscle damage. Antioxidants (Basel).

[5] Sonkodi B, Bardoni R, Hangody L, Radák Z, Berkes I (2021). Does compression sensory axonopathy in the proximal tibia contribute to noncontact anterior cruciate ligament injury in a causative way? A new theory for the injury mechanism. Life.

[6] Ali N, Rouhi G (2010). Barriers to predicting the mechanisms and risk factors of non-contact anterior cruciate ligament injury. Open Biomed Eng J.

[7] Kobayashi H, Kanamura T, Koshida S, Miyashita K, Okado T, Shimizu T (2010). Mechanisms of the anterior cruciate ligament injury in sports activities: a twenty-year clinical research of 1700 athletes. J Sports Sci Med.

[8] Koga H, Nakamae A, Shima Y, Iwasa J, Myklebust G, Engebretsen L (2010). Mechanisms for noncontact anterior cruciate ligament injuries: knee joint kinematics in 10 injury situations from female team handball and basketball. Am J Sports Med.

[9] McNair PJ, Marshall RN, Matheson JA (1990). Important features associated with acute anterior cruciate ligament injury. N Z Med J.

[10] Boden BP, Dean GS, Feagin JA, Garrett WE (2000). Mechanisms of anterior cruciate ligament injury. Orthopedics.

[11] Fauno P, Wulff JB (2006). Mechanism of anterior cruciate ligament injuries in soccer. Int J Sports Med.

[12] Sonkodi B (2021). Delayed onset muscle soreness (DOMS): the repeated bout effect and chemotherapy-induced axonopathy may help explain the dying-back mechanism in amyotrophic lateral sclerosis and other neurodegenerative diseases. Brain Sci.

[13] Vila-Cha C, Hassanlouei H, Farina D, Falla D (2012). Eccentric exercise and delayed onset muscle soreness of the quadriceps induce adjustments in agonist-antagonist activity, which are dependent on the motor task. Exp Brain Res.

[14] Marathamuthu S, Selvanayagam VS, Yusof A. Contralateral effects of eccentric exercise and DOMS of the plantar flexors: evidence of central involvement. Res Q Exerc Sport. 2020:1–10. 10.1080/02701367.2020.1819526 32976088

[15] Hedayatpour N, Izanloo Z, Falla D (2018). The effect of eccentric exercise and delayed onset muscle soreness on the homologous muscle of the contralateral limb. J Electromyogr Kinesiol.

[16] Bardoni R, Torsney C, Tong CK, Prandini M, MacDermott AB (2004). Presynaptic NMDA receptors modulate glutamate release from primary sensory neurons in rat spinal cord dorsal horn. J Neurosci.

[17] Relph N, Herrington L, Tyson S (2014). The effects of ACL injury on knee proprioception: a meta-analysis. Physiotherapy.

[18] Johansson H, Sjolander P, Sojka P (1991). A sensory role for the cruciate ligaments. Clin Orthop Relat Res.

[19] Schutte MJ, Dabezies EJ, Zimny ML, Happel LT (1987). Neural anatomy of the human anterior cruciate ligament. J Bone Joint Surg Am.

[20] Kim KO, Sim JA, Choi JU, Lee BK, Park HG (2020). The effect of interleukin-8 in the early stage after anterior cruciate ligament reconstruction with remnant preservation. Knee Surg Relat Res.

[21] Georgoulis AD, Pappa L, Moebius U, Malamou-Mitsi V, Pappa S, Papageorgiou CO (2001). The presence of proprioceptive mechanoreceptors in the remnants of the ruptured ACL as a possible source of re-innervation of the ACL autograft. Knee Surg Sports Traumatol Arthrosc.

[22] Ochi M, Adachi N, Deie M, Kanaya A (2006). Anterior cruciate ligament augmentation procedure with a 1-incision technique: anteromedial bundle or posterolateral bundle reconstruction. Arthroscopy.

[23] Grassi A, Agostinone P, Di Paolo S, Lucidi GA, Macchiarola L, Bontempi M (2021). Knee position at the moment of bone bruise could reflect the late phase of non-contact anterior cruciate ligament injury rather than the mechanisms leading to ligament failure. Knee Surg Sports Traumatol Arthrosc.

[24] Gali JC, Resina AF, Pedro G, Neto IA, Almagro MA, da Silva PA (2014). Importance of anatomically locating the infrapatellar branch of the saphenous nerve in reconstructing the anterior cruciate ligament using flexor tendons. Rev Bras Ortop.

[25] Pagnani MJ, Warner JJ, O'Brien SJ, Warren RF (1993). Anatomic considerations in harvesting the semitendinosus and gracilis tendons and a technique of harvest. Am J Sports Med.

[26] Boon JM, Van Wyk MJ, Jordaan D (2004). A safe area and angle for harvesting autogenous tendons for anterior cruciate ligament reconstruction. Surg Radiol Anat.

[27] Figueroa D, Calvo R, Vaisman A, Campero M, Moraga C (2008). Injury to the infrapatellar branch of the saphenous nerve in ACL reconstruction with the hamstrings technique: clinical and electrophysiological study. Knee.

[28] Mirzatolooei F, Pisoodeh K (2012). Impact of exploration of sensory branches of saphenous nerve in anterior cruciate ligament reconstructive surgery. Arch Iran Med.

[29] McHugh MP, Connolly DA, Eston RG, Gleim GW (1999). Exercise-induced muscle damage and potential mechanisms for the repeated bout effect. Sports Med.

[30] Nosaka K, Sakamoto K, Newton M, Sacco P (2001). How long does the protective effect on eccentric exercise-induced muscle damage last?. Med Sci Sports Exerc.

[31] Hortobagyi T, Lambert NJ, Hill JP (1997). Greater cross education following training with muscle lengthening than shortening. Med Sci Sports Exerc.

[32] Howatson G, van Someren KA (2007). Evidence of a contralateral repeated bout effect after maximal eccentric contractions. Eur J Appl Physiol.

[33] Chen TC, Chen HL, Lin MJ, Yu HI, Nosaka K (2016). Contralateral repeated bout effect of eccentric exercise of the elbow flexors. Med Sci Sports Exerc.

[34] Hyldahl RD, Chen TC, Nosaka K (2017). Mechanisms and mediators of the skeletal muscle repeated bout effect. Exerc Sport Sci Rev.

[35] Kuphal KE, Fibuch EE, Taylor BK (2007). Extended swimming exercise reduces inflammatory and peripheral neuropathic pain in rodents. J Pain.

[36] Sonkodi B, Kopa Z, Nyirady P (2021). Post orgasmic illness syndrome (POIS) and delayed onset muscle soreness (DOMS): do they have anything in common?. Cells.

[37] Andriacchi TP, Favre J (2014). The nature of in vivo mechanical signals that influence cartilage health and progression to knee osteoarthritis. Curr Rheumatol Rep.

[38] Tayfur B, Charuphongsa C, Morrissey D, Miller SC (2021). Neuromuscular function of the knee joint following knee injuries: does it ever get back to normal? A systematic review with meta-analyses. Sports Med.

[39] Hortobagyi T, Garry J, Holbert D, Devita P (2004). Aberrations in the control of quadriceps muscle force in patients with knee osteoarthritis. Arthritis Rheum.

[40] Andriacchi TP, Koo S, Scanlan SF (2009). Gait mechanics influence healthy cartilage morphology and osteoarthritis of the knee. J Bone Joint Surg Am.

[41] Onate J, Herman D, Grooms DR, Sutton Z, Wilkerson G. Neuroscience principles for ACL rehabilitation and reinjury risk reduction. 2019; 2019.

[42] Wiggins AJ, Grandhi RK, Schneider DK, Stanfield D, Webster KE, Myer GD (2016). Risk of secondary injury in younger athletes after anterior cruciate ligament reconstruction: a systematic review and meta-analysis. Am J Sports Med.

[43] Friel NA, Chu CR (2013). The role of ACL injury in the development of posttraumatic knee osteoarthritis. Clin Sports Med.

[44] Melnyk M, Faist M, Gothner M, Claes L, Friemert B (2007). Changes in stretch reflex excitability are related to "giving way" symptoms in patients with anterior cruciate ligament rupture. J Neurophysiol.

[45] Horslen BC, Zaback M, Inglis JT, Blouin JS, Carpenter MG (2018). Increased human stretch reflex dynamic sensitivity with height-induced postural threat. J Physiol.

[46] Sonnery-Cottet B, Saithna A, Quelard B, Daggett M, Borade A, Ouanezar H (2019). Arthrogenic muscle inhibition after ACL reconstruction: a scoping review of the efficacy of interventions. Br J Sports Med.

[47] Smeets A, Verschueren S, Staes F, Vandenneucker H, Claes S, Vanrenterghem J (2021). Athletes with an ACL reconstruction show a different neuromuscular response to environmental challenges compared to uninjured athletes. Gait Posture.

[48] Lin SH, Cheng YR, Banks RW, Min MY, Bewick GS, Chen CC (2016). Evidence for the involvement of ASIC3 in sensory mechanotransduction in proprioceptors. Nat Commun.

[49] Woo SH, Lukacs V, de Nooij JC, Zaytseva D, Criddle CR, Francisco A (2015). Piezo2 is the principal mechanotransduction channel for proprioception. Nat Neurosci.

[50] Niibori M, Kudo Y, Hayakawa T, Ikoma-Seki K, Kawamata R, Sato A (2020). Mechanism of aspirin-induced inhibition on the secondary hyperalgesia in osteoarthritis model rats. Heliyon.

[51] Ikeuchi M, Kolker SJ, Burnes LA, Walder RY, Sluka KA (2008). Role of ASIC3 in the primary and secondary hyperalgesia produced by joint inflammation in mice. Pain.

[52] Vralsted VC, Price MP, Du J, Schnizler M, Wunsch AM, Ziemann AE (2011). Expressing acid-sensing ion channel 3 in the brain alters acid-evoked currents and impairs fear conditioning. Genes Brain Behav.

[53] Venugopal S, Hamm TM, Crook SM, Jung R (2011). Modulation of inhibitory strength and kinetics facilitates regulation of persistent inward currents and motoneuron excitability following spinal cord injury. J Neurophysiol.

[54] Rice DA, McNair PJ, Lewis GN, Dalbeth N (2014). Quadriceps arthrogenic muscle inhibition: the effects of experimental knee joint effusion on motor cortex excitability. Arthritis Res Ther.

[55] Makkar SR, Zhang SQ, Cranney J (2010). Behavioral and neural analysis of GABA in the acquisition, consolidation, reconsolidation, and extinction of fear memory. Neuropsychopharmacology.

[56] Ievglevskyi O, Isaev D, Netsyk O, Romanov A, Fedoriuk M, Maximyuk O (2016). Acid-sensing ion channels regulate spontaneous inhibitory activity in the hippocampus: possible implications for epilepsy. Philos Trans R Soc Lond B Biol Sci.

[57] Kim WB, Cho JH (2020). Encoding of contextual fear memory in hippocampal-amygdala circuit. Nat Commun.

[58] Ardern CL, Osterberg A, Tagesson S, Gauffin H, Webster KE, Kvist J (2014). The impact of psychological readiness to return to sport and recreational activities after anterior cruciate ligament reconstruction. Br J Sports Med.

[59] Ohji S, Aizawa J, Hirohata K, Ohmi T, Mitomo S, Koga H (2021). Injury-related fear in athletes returning to sports after anterior cruciate ligament reconstruction—a quantitative content analysis of an open-ended questionnaire. Asia Pac J Sports Med Arthrosc Rehabil Technol.

[60] Kakavas G, Malliaropoulos N, Pruna R, Traster D, Bikos G, Maffulli N (2020). Neuroplasticity and anterior cruciate ligament injury. Indian J Orthop.

[61] Kakavas G, Malliaropoulos N, Bikos G, Pruna R, Valle X, Tsaklis P (2021). Periodization in anterior cruciate ligament rehabilitation: a novel framework. Med Princ Pract.

[62] Tufano JJ, Brown LE, Coburn JW, Tsang KK, Cazas VL, LaPorta JW (2012). Effect of aerobic recovery intensity on delayed-onset muscle soreness and strength. J Strength Cond Res.

